# Intensive Care Admission and Early Neuro-Rehabilitation. Lessons for COVID-19?

**DOI:** 10.3389/fneur.2020.00880

**Published:** 2020-08-25

**Authors:** Alessandro Pincherle, Jane Jöhr, Lisa Pancini, Letizia Leocani, Laura Dalla Vecchia, Philippe Ryvlin, Nicholas D. Schiff, Karin Diserens

**Affiliations:** ^1^Acute Neuro-Rehabilitation Unit and Neurology Unit, Department of Clinical Neurosciences, Lausanne University Hospital-CHUV, Lausanne, Switzerland; ^2^Neurology Unit, Department of Medicine, Hopitaux Robert Schuman—Luxembourg, Luxembourg, Luxembourg; ^3^Departments of Cardiac and Pulmonary Rehabilitation, IRCSS Istituto Clinico Scientifico Maugeri, Milan, Italy; ^4^Department of Neuro-Rehabilitation, Hospital San Raffaele, University Vita Salute, Milan, Italy; ^5^Feil Family Brain and Mind Research Institute, Weill Cornell Medicine, New York, NY, United States

**Keywords:** COVID-19, early rehabilitation, ICU, immobilization, mechanical ventilation, neurological complications

## Abstract

Coronavirus disease 2019 (COVID-19) requires admission to intensive care (ICU) for the management of acute respiratory distress syndrome in about 5% of cases. Although our understanding of COVID-19 is still incomplete, a growing body of evidence is indicating potential direct deleterious effects on the central and peripheral nervous systems. Indeed, complex and long-lasting physical, cognitive, and functional impairments have often been observed after COVID-19. Early (defined as during and immediately after ICU discharge) rehabilitative interventions are fundamental for reducing the neurological burden of a disease that already heavily affects lung function with pulmonary fibrosis as a possible long-term consequence. In addition, ameliorating neuromuscular weakness with early rehabilitation would improve the efficiency of respiratory function as respiratory muscle atrophy worsens lung capacity. This review briefly summarizes the polymorphic burden of COVID-19 and addresses possible early interventions that could minimize the neurological and systemic impact. In fact, the benefits of early multidisciplinary rehabilitation after an ICU stay have been shown to be advantageous in several clinical conditions making an early rehabilitative approach generalizable and desirable to physicians from a wide range of different specialties.

## Introduction

Coronavirus disease 2019 (COVID-19), caused by the severe acute respiratory syndrome coronavirus 2 (SARS-CoV-2), is a major burden on Intensive Care Units (ICU) because of the high number of patients eventually requiring respiratory support measures. Although most COVID-19 patients are asymptomatic or experience mild illness, ~15% become severely ill, requiring oxygen therapy. A further 5% are admitted to an ICU, where they require invasive ventilation for acute respiratory distress syndrome (ARDS) (1).

Regardless of the underlying pathology, prolonged ICU stay frequently involves sedation and immobilization (often in a prone position). This is associated with musculoskeletal, pulmonary, cardiovascular, immunological, endocrine, and metabolic complications (2). Musculoskeletal consequences are especially relevant and include muscle atrophy, decreased strength, reduced protein synthesis, joint contractures, bone density decrease, and pressure ulcers. Nearly 50% of ICU patients show critical illness-associated neuromuscular abnormalities (3). If ventilatory support is maintained for longer than 14 days, a tracheotomy is recommended (4). As a result, a high proportion of COVID-19 patients undergo this procedure in their extended ICU stay.

**Figure 1:**
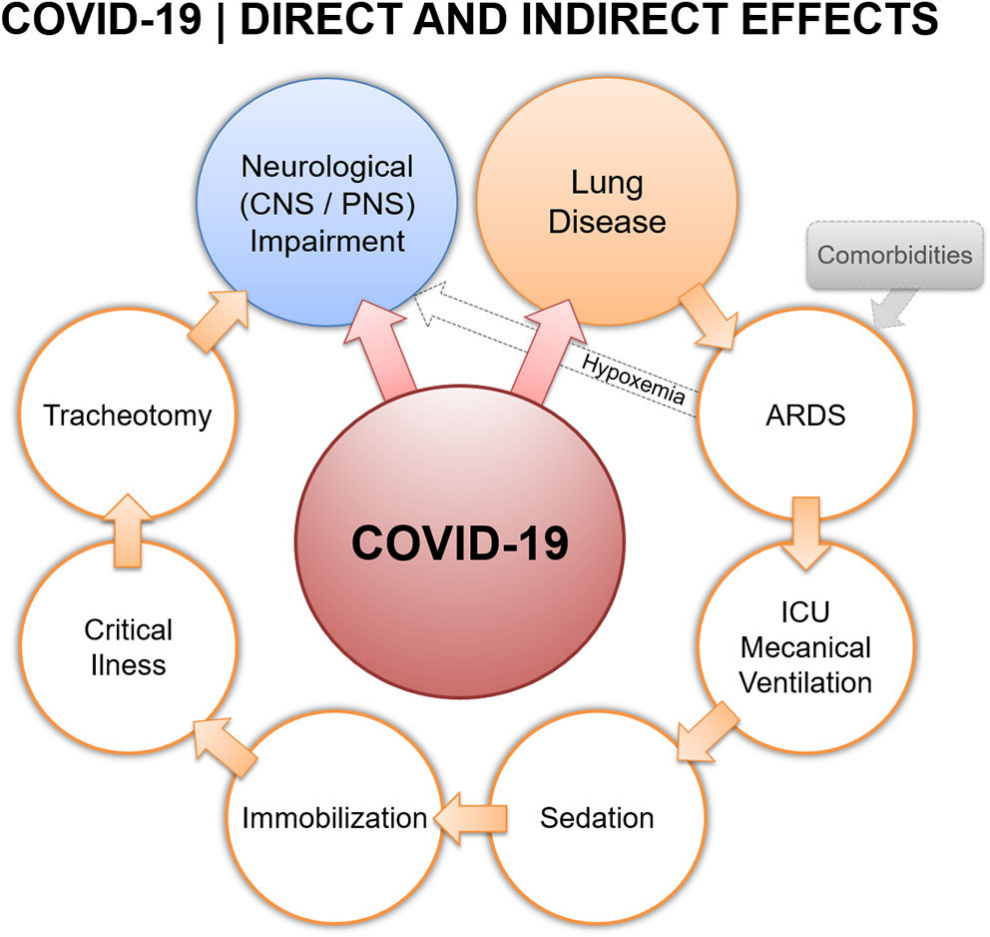
Covid-19 direct and indirect effects.

The complexity of ICU patient management is compounded if the underlying disease touches the central and/or peripheral nervous system (CNS, PNS). In the case of COVID-19, emerging preliminary evidence points toward significant neurological involvement (5–8). Actually, possible nervous system infection could occur by direct entry of the virus via the cribriform plate (8) or, through systemic circulatory dissemination following infection of the lungs. COVID-19 patients admitted to ICU should therefore be considered as especially critical given the potential nervous system involvement. A higher risk of developing transient or persistent neuromuscular and/or neurological sequelae/deficits is consequently conceivable (Figure 1).

In this respect, early (defined as during and immediately after ICU discharge) rehabilitative interventions are fundamental in reducing possible added neurological burden to a disease that already greatly affects lung function, potentially causing pulmonary fibrosis in the long-term. In addition, managing neuromuscular weakness would improve the efficiency of respiratory function, as respiratory muscle atrophy worsens lung capacity.

Here, we first briefly summarize the current knowledge on the repercussions of COVID-19, mainly focusing on the neurological manifestations and complications. We compiled the available literature by performing computer searches of English-language databases (Medline, PubMed Central, Google Scholar) combining the relevant keywords (“COVID-19,” “Coronavirus,” “early rehabilitation,” “neurological complications”) up to 1st May 2020. Then, we address early rehabilitative interventions that could minimize the neurological impact of COVID-19. Many lessons can be learnt from the cumulative experience of early rehabilitation strategies applied in the acute stage on severely and critically ill patients. However, it is pertinent to point out that this knowledge continues to evolve as new data is being shared regularly, and new recommendations may be provided as more evidence emerges.

## Why COVID-19 Impairs Recovery

Several features, comorbidities, and complications of COVID-19 are associated with adverse effects on multiples organs and systems other than the respiratory system, which may then lead to high levels of physical, cognitive, and functional impairment. Rehabilitation treatment plans for patients with COVID-19 or recovering from it, ought to take into consideration these implications to restore impaired functions and prevent long-term consequences.

### CNS Involvement

Adverse cerebrovascular events have been reported in COVID-19 patients who developed severe respiratory complications (1). In one study, hypoxic/ischemic encephalopathy was reported in ~20% of 113 patients who all died from COVID-19 (9). Additionally, a recent investigation from China found that among 214 COVID-19 patients, approximately one third experienced neurological manifestations, including acute cerebrovascular disease, and impaired consciousness (10). Hemorrhagic necrotizing encephalopathy and medio-temporal epileptic encephalitis have also been reported (11, 12). In a French consecutive cases series of 58 severe acute-COVID-19 patients, encephalopathy with prominent agitation, confusion, and corticospinal tract signs were observed in almost two thirds of cases. Eight out of 13 patients who received a brain MRI in this series showed an enhancement of the leptomeningeal spaces. Furthermore, in the 11 patients who underwent perfusion imaging, all showed bilateral frontotemporal hypoperfusion (13). Importantly in this cohort, enduring dysexecutive symptoms accompanied recovery, identifying a target for late rehabilitation effort and follow-up treatment.

The link between COVID-19 and cerebrovascular disease remains controversial but many findings suggest that ischemic stroke occurs in the context of a systemic, highly pro-thrombotic state. An increase in the number of large vessel occlusion malignant strokes has been independently reported (14–16), combined with a higher number of life-threatening thrombotic complications (17). Post-mortem observations have demonstrated multi-organ endothelitis with significant micro-vascular impairment (18).

Although based on a limited amount of data, some evidence suggests that coronaviruses may cause damage to the dopaminergic system. A selective affinity of coronaviruses for the basal ganglia and limbic system has been reported in rodent models of encephalitis induced by intranasal inoculation (19). Specifically, intraneuronal transport was posited as the spreading mechanism of the viruses (20). In humans, high anti-coronavirus antibody titers were observed in the cerebrospinal fluid of Parkinson's disease patients (21). Furthermore, using electron-microscopy the virus was detected in frontal-lobe tissue (22). Prolonged confusion after sedation withdrawal and impaired consciousness have also been described in COVID-19 patients (23). This is not surprising given that functional disturbance of the forebrain systems (frontal/prefrontal, cortical-striato-pallidal, and thalamocortical loop systems) are known to be associated with cognitive-motor dissociation in severe brain injuries. Cognitive-motor dissociation is characterized by blocked motor preparation and action (24). Of note, the possibility that dopaminergic systems may become deregulated suggests that in some COVID-19 patients, altered consciousness may reflect a functional akinetic mutism (25); in such instances a more marked alteration of resting metabolism could be diagnostic or the direct evaluation of altered dopaminergic transmission (26). In summary, COVID-19 can induce neurological sequelae by attacking the CNS in a multifaceted way. This includes vascular, inflammatory, and/or direct neuronal injury. Furthermore, this neurological intrusion may be clinically silent because of sedation and avoidance of certain diagnostic procedures to reduce the risk of cross infection including lumbar puncture, brain imaging, and electromyography/nerve conduction velocity.

### PNS Involvement and Critical Care Illness

Several cases of post-COVID-19 acute polyneuropathy have been reported (7, 27–32) with electro-clinical characteristics of Guillain Barré syndrome. These include acute inflammatory demyelinating and motor-sensory axonal subtypes. In the largest case series to date, Toscano et al. (32) demonstrated that of five Italian patients, an axonal variant was observed in three of them and a demyelinating form in two. Furthermore, two cases of Miller-Fisher variant, a Guillain Barré subtype with cranial nerve involvement, have also been described (33). Mao et al. (10) first proposed that anosmia and ageusia in COVID-19 patients reflected involvement at the cranial nerve level. In line with this theory, a large case-controlled study of COVID-19 patients presenting with smell and/or taste disorders, found that <15% reported concomitant nasal obstruction indicating a primary dysfunction of the olfactory tract (34).

Data are still lacking to prove a specific association between critical illness–related myopathy or neuropathy (CRIMYNE) and COVID-19. However, data from the severe acute respiratory syndrome (SARS) outbreak in 2003 indicated that myopathies with severe muscle wasting and myalgias, were very frequently associated with coronavirus infections (35). As previously stated, nearly 50% of ICU patients present critical illness-associated neuromuscular abnormalities (3). It is therefore arguable that COVID-19 patients are especially at risk of PNS damage.

### Respiratory Impairment and Tracheotomy

COVID-19 causes varying degrees of lung complication. These range from mild to severe pneumonia, ARDS, and sepsis. In mild or uncomplicated illness, patients present with symptoms of upper respiratory tract viral infection. These symptoms include mild fever, a dry cough, sore throat, nasal congestion, malaise, headache, and muscle pain. In severe pneumonia, fever is associated with serious dyspnea, respiratory distress, tachypnea (> 30 breaths/min), and hypoxia (SpO2 <90% on room air) (36). Chest imaging results may be normal in early or mild disease, however, in patients requiring hospitalization, 69% have abnormal chest X-Rays at admission (37). The most frequent findings on X-Ray and CT scans are airspace opacities including ground-glass opacity or consolidation. Distribution is most often bilateral, peripheral, and lower zone predominant (37). In some patients, major alveolar damage results in hypoxemic acute respiratory failure (ARF), requiring mechanical ventilation and ICU admission. Moreover, respiratory viral infections predispose to co-infections resulting in increased disease severity and mortality. Zhou et al. (38) showed that 50% of patients who died from COVID-19 had secondary bacterial infections. Additionally, Chen et al. (9) recorded bacterial and fungal co-infections in COVID-19 patients. Although 71% of patients admitted with COVID-19 receive antibiotics, no information is available on the antimicrobial sensitivities of the organisms identified or on the type and duration of antimicrobial treatment (39).

Chronic obstructive pulmonary disease (COPD) is a risk factor for severe COVID-19. Many patients with COPD have underlying chronic bacterial infections prior to the SARS-CoV-2 infection; however, this important information is not being reported. Given the neuro-invasive potential of SARS-CoV-2 and the peculiar severity of respiratory failure observed in COVID-19 patients, researchers have suggested involvement of the CNS respiratory centers (40). However, to date, there are no data proving SARS-CoV-2 invasion of brainstem dorsal root neurons; furthermore, recovery is typically longer and more difficult in patients having neuroinflammation or neurodegeneration in these areas than in COVID-19 disease (41). While the majority of patients recover from pneumonia without any lasting lung damage, the lasting effects of COVID-19-associated pneumonia may be drastic. Following recovery from acute COVID-19, lung injury may lead to shortness of breath that takes months to get better. Indeed, COVID-19 patients who recover from ARDS may have lasting pulmonary scarring/fibrosis.

In a typical non-COVID-19 ICU patient cohort, early tracheotomy is often performed for critically-ill ventilated patients based on several arguments, including decreasing the duration of mechanical ventilation and ICU stay. However, the ensuing reduction in mortality rate described in several studies, remains a matter of controversy (42–45). It is widely accepted that tracheostomy presents various drawbacks with delayed effects. These include swallowing disorders, mucous plugs, and granulations. Cuff inflation, which causes irritation or damage to the tracheal mucosa, is related to these consequences. In addition, the lack of air-flow induces deafferentation of the oropharyngeal region, which perturbs the swallowing process (46).

Tracheostomy patients requiring repeated aspirations might need continual monitoring and significant support, which incurs additional costs. Rapid and safe weaning of tracheostomy patients is therefore an important goal. Several recent studies have confirmed that a multidisciplinary approach significantly reduces weaning time in acute care (47).

### Cognitive Impairment

Neurocognitive impairments in COVID-19 patients have not yet been widely reported. Nonetheless, the tendency of SARS-CoV-2 to invade and disseminate into the CNS through a synapse-connected route, similarly to other coronaviruses, may lead to severe neurological consequences (48, 49). A recent systematic review suggested that a substantial proportion of patients with severe COVID-19 were highly likely to experience a impaired mental status (5). Furthermore, recent findings of a retrospective study of 214 COVID-19 patients described various neurological manifestations. Among the severe cases, impaired consciousness was observed in 14.8% (10). Neuro-radiological investigation of the first meningitis/encephalitis case associated with SARS-CoV-2 demonstrated inflammation in brain structures supporting memory functions, namely the medial temporal lobe including the hippocampus (12). Early case reports from Italy highlighted the importance of recognizing the development of encephalopathy both as a risk during hospital stay and, as a symptom of COVID-19 (50). In addition, older age and preexisting cognitive conditions were highlighted as enhancing the risk of developing encephalopathy during acute infection and critical illness. Indeed, neurological dysfunction, including delirium and cognitive impairment, is extremely common following critical illness and its pharmacological management (51, 52).

The majority of the literature concludes that several mechanisms such as hypoxemia, glucose dysregulation, and the effects of sedation contribute to development of neurological dysfunction. Studies regarding cognitive outcomes following critical illness report damage over a range of domains including attention, memory, processing speed, and executive function (52–54). A large cohort study of 821 patients in medical and surgical ICUs estimated a high risk of long-term cognitive impairment following critical illness (55). They reported a significant positive correlation between longer duration of delirium with worse global cognition and executive function scores at 3 and 12 months. Moreover, deficits in executive abilities are prominent in patients suffering from conditions such as ARDS, which include symptoms resulting from hypoxemia. This is coherent with the evidence suggesting that structures within the frontal circuits are sensitive to hypoxia (56).

Literature specifically regarding the long-term outcomes in ARDS survivors reported that 1 year after discharge, the majority experienced neuropsychological disabilities including impaired memory, attention, concentration, and mental-processing speed and a global intellectual decline. Prevalence ranged from 25 (57) to 78% depending on the severity of the ARDS (51). A prospective multicenter study in 174 ARDS patients found that at 12 months, 25% of survivors had cognitive impairment in their executive functions, language, immediate, and delayed memory, verbal reasoning and concept formation, and attention and working memory. However, 36% showed significant improvement at 6 months (58). In another study, 82 ARDS survivors self-reported a high prevalence of depressive symptoms and a low prevalence of memory deficits 6–48 months after ICU discharge (59).

As new data on the novel coronavirus SARS-CoV2 continue to reveal its involvement in the CNS, primary deficits in executive functions, attention, and memory may be expected and should be addressed immediately in the acute phase. In this respect, early (unpublished) clinical data from post-acute COVID-19 infected patients in our Swiss hospital are consistent with the expectations. They exposed that executive deficits ranged from light to severe, that attention disturbances were observed in all patients, and more than two-thirds presented memory alteration. Furthermore, from our clinical neurological examination of acute COVID-19 patients in the ICU, severe forms of akinesia were frequently encountered. This may lead to clinical underestimation of conscious awareness in the acute phase, a condition described as cognitive-motor dissociation (60). Indeed, in cases of severely impaired motor output, a patient's cognitive capacity to interact may be hampered and misdiagnosed as reflecting forms of severe altered consciousness carrying unfavorable prognosis (61, 62).

## Rehabilitation Strategies to Enhance Recovery After COVID-19

**Figure 2:**
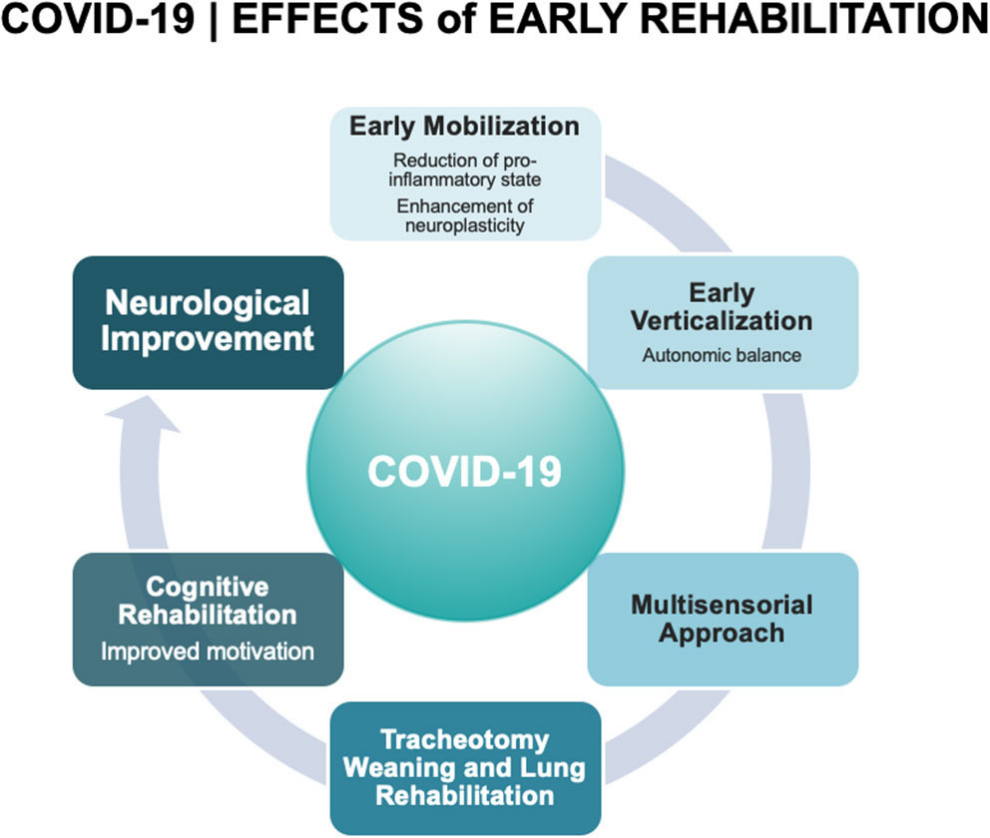
Covid-19 effects of early rehabilitation.

Rehabilitation is a complex intervention that focuses on reducing disability, decreasing dependency, and increasing the quality of life. Early rehabilitative interventions following COVID-19 could be similar to those of patients with severe brain injuries or critical illnesses also requiring a prolonged ICU stay. In this respect, they should target recovery of the respiratory system and cardiovascular reconditioning but also recovery of mobility, functioning, and cognition (Figure 2). Rehabilitative intervention programs should be implemented according to the framework of the International Classification of Functioning, Disability, and Health (63, 64), which integrates an individualized treatment plan addressing personal functioning, disease and disability. This promotes and optimizes functional independence thus maximizing a return to participation in society.

### Early Mobilization

Muscle deconditioning occurs very early with bed rest, involving a decline in muscle mass, strength, and aerobic efficiency. ICU-acquired weakness is found in in ~25% of patients (65). This worsens acute morbidity and increases the mortality risk at 1 year (66). Mechanically ventilated patients warrant close attention because of the increased risk of developing ICU-acquired weakness (67). In this respect, COVID-19 patients needing ventilator support for extended periods should be considered especially at risk. Evidence of benefit from early mobilization and physiotherapy comes from numerous randomized controlled trials, systematic reviews, and recommendations (68, 69) and is proven to be a safe and effective intervention. Early mobilization (70), can be initiated during the mechanical ventilation to counteract ICU-acquired weakness. However, an agreed method of early mobilization in mechanically ventilated patients is currently lacking, thus limiting reproducibility and dissemination of shared protocols (71). While official guidelines suggest the use of early mobilization protocols, they do not recommend a specific one (72) and international practices are heterogeneous (61). Unfortunately, there is also limited awareness of the clinical benefits of early mobilization and physiotherapy techniques and when used, disagreement on the sustainable maximal level of activity in these critically-ill patients. However, several factors including multidisciplinary rounds, setting daily goals for patients, day-to-day availability of dedicated physiotherapists, and an adequate nurse/patient ratio are becoming significantly associated with the practice of early mobilization in ICUs.

In our Swiss University Hospital, we adopt a pre-specified procedure for early mobilization with clear entry and exit points. In the ICU, mobilization of mechanically ventilated patients is achieved using MOTOmed Letto® (Reck & Co. GmbH, Germany; an automatic system for leg movement in a supine position, mimicking a bicycle, allowing passive, active, or assisted mobilization) and Erigo® (Hocoma AG, Switzerland; a tilting table with an integrated leg movement system, allowing progressive verticalization of the patient, adjustable to the patient's needs, and possibilities) as soon as cardiovascular stability of the patient is attained. Many animal and human studies suggest that intermittent exposure to gravity throughout long periods of bed rest is sufficient to prevent deconditioning (73) and improve outcome after awakening from a coma (74). Verticalization is now integrated into a neuro-sensorial approach in acute neuro-rehabilitation and improves the results of tracheostomy weaning (75). In addition, a multidisciplinary approach (physiotherapists, nurses, physicians) allows a rapid and pertinent adaptation to the different stages as it takes into account the great variability in neurological deficits and the considerable intra-individual requirements for patient management (75). A prospective randomized study on patients with severe brain injuries showed that mobilization with the lower-body ergometer MOTOmed®, was able to prevent polyneuromyopathy in critical-care illness and to improve awareness in disorders of consciousness. Of note, the use of the robot Erigo® proved to be safer in patients with subarachnoid hemorrhage as it has no effect on the production of catecholamines (76). Futhermore, mobilization has potentiation effect on arousal that may support cognitive recovery as well (77).

### Neurosensory Stimulation Approach

The restricted mobility, impaired communication, and social isolation that COVID-19 patients experience in the ICU due to mechanical ventilation may lead to severe sensory deprivation. Environmental (i.e., sensory) deprivation is described as a reduction in variety and intensity of sensory input (78) and can slow down the recovery and development of CNS function (79). Sensory deficits may have additional negative effects of majorly stressing the body and so altering its physiological balance (80). A rationale for treatment is to enrich the environment, promoting the brain's plasticity processes, thereby enabling organizational, and functional modifications. Interventions use multisensory-stimulation programs, which promote arousal, and behavioral responsiveness from controlled exposure to environmental or sensory-specific stimuli (81). Sensory stimuli include visual, auditory, tactile, olfactory, gustatory, and proprioceptive stimulation that can vary considerably in form, intensity, and number of modalities but are typically variations of multisensory stimulation, including the presentation of stimuli that are structured, meaningful, multimodal, familiar, and with emotional content. This maximizes the probability of cognitive engagement (82). For instance, affective auditory stimulation can be achieved by providing information about a time and place, using the patient's favorite music, playing the voice of a loved one, talking to the patient about happy daily events in his/her family or pleasant memories and enjoyable experiences; a tactile and proprioceptive stimulation can be applied by massaging the patient's hands and legs and performing passive range-of-motion activities several times; a visual stimulation can be applied by using a picture of a family member, a family film, or a picture with high positive valence; an olfactory stimulation can be applied using aromatic stimuli including the patient's favorite aromas; a gustatory stimulation can be applied by placing different kinds of food and flavors on the patient's tongue with a cotton bud.

In the clinic, multisensory stimulation is the core of basal stimulation, a therapeutic concept developed by Andreas Fröhlich (83) and subsequently transferred into nursing. Basal stimulation aims to provide a structured and accessible perceptual experience through stimulation of the body and its movements. In addition, it aims to develop an individual, non-verbal form of communication with people whose own activity is limited by their lack of mobility and whose ability to perceive and communicate is significantly impaired. Sensory stimulation is a non-invasive, safe, inexpensive, and simple-to-apply rehabilitation approach, which has been widely studied in patients with severe brain injuries experiencing alterations in consciousness (84). Despite the lack of high-quality clinical trials, the literature suggests that applying a sensory stimulation protocol enhances the recovery process and improves outcomes in severely brain-injured patients (84–86).

### Cognitive Rehabilitation

Alongside respiratory physiotherapy and functional rehabilitation, additional cognitive rehabilitation may be required for COVID-19 patients who present neuropsychological alterations in cognitive performance in the acute and immediate post-acute phases. Formal rehabilitation pathways, comparable to those used in stroke and traumatic brain injury patients, do not yet exist for survivors of acute COVID-19 (87, 88). However, as awareness of COVID-19-induced cognitive impairments grows, rehabilitation strategies should also focus on cognitive recovery.

Cognitive rehabilitation is a broad term referring to therapeutic approaches that address the cognitive deficits caused by lesions or illnesses affecting the brain's optimal functionality. Most methods use either a restorative or compensatory approach (88). The restorative approach aims at rehabilitating cognitive functions by reinforcing, strengthening, or re-establishing previously learned patterns of behavior. It includes repeated exercise of standardized cognitive tests of increasing difficulty that target specific cognitive domains (e.g., selective attention, memory for new information). In contrast, the compensatory approach uses alternative strategies (e.g., internal residual strengths or external compensatory mechanisms including environmental structure and support) that compensate for the decline in cognitive function. Several principles underpin its process and effectiveness (89). Therapeutic interventions have shown greater benefit when integrated as part of a multidisciplinary rehabilitative approach (90) and tailored to the individual needs with goals regularly reassessed (91). Additionally, if interventions are of increasing intensity (92) and begin as soon after injury as possible (89), they are more likely to be successful. In patients with acquired cerebral lesions including traumatic brain injury and stroke, successful cognitive rehabilitation has previously been demonstrated. Systematic reviews on evidence-based cognitive rehabilitation emphasize the importance of functional, patient-centered outcomes. They advise developing individualized and motivational interventions documented by more subjective outcome measures (93).

As previously hypothesized, patients with severe, and critical COVID-19 may present with disturbances primarily in executive functions including severe akinesia (as seen in cognitive-motor dissociation), as well as in attention and memory. Moreover, attention and memory deficits may be exacerbated following periods of delirium. This can lead to additional disturbances in other complex cognitive functions, such as interpersonal communication skills. Similar to patients with severe brain injuries, the acute rehabilitative treatment of COVID-19 patients should aim to improve attention and stimulate the networks responsible for conscious perception and environmental interaction. Promoting motivational stimulation (94, 95) and increasing sensory input (84) may increase adequate goal-oriented behaviors, enhance the recovery process, and minimize the risk of functional disability (88). Post-acute rehabilitation of COVID-19 patients should focus on interventions that improve everyday functioning. They should directly apply compensatory strategies to functional contexts while considering appropriate infection-control measures. This may necessitate the use of remote support services such as tele-rehabilitation, virtual care platforms, and communication devices (96).

### Respiratory Support and Physiotherapy

Severely and critically ill patients suffer varying degrees of dysfunction, especially respiratory insufficiency during the acute and recovery stages. The goal of early rehabilitation intervention is to reduce breathing difficulties, relieve symptoms, ease anxiety and depression, and lower the incidence of complications.

Rehabilitation interventions in severely or critically ill COVID-19 patients can only begin when the minimum clinical stability has been achieved. Treatments should be immediately withdrawn in cases of high fever, worsening dyspnea, a respiratory rate > 30 breaths/minute, pulse oximetry <93% on oxygen therapy or requiring FiO2 > 50% during non-invasive ventilation (NIV), positive end expiratory pressure (PEEP)/continuous positive airway pressure (CPAP) >10 cm H2O, respiratory distress, arterial hypertension, brady- or tachycardia, intercurrent arrhythmias, shock, deep sedation, or evidence of radiological lesion progression (>50%) within 24–48 h. Rehabilitation therapy in these cases mainly includes position management, respiratory training, and mild physical exercise. Frequent changes of posture, passive mobilization, and/or neuromuscular electrical stimulation should be planned especially in the unconscious patient (97). In addition, evaluation of peripheral muscle strength trends [by the Medical Research Council [MRC] scale and dynamometers] should be recorded as soon as practicable. Airway clearance techniques are not recommended in the acute phase. Indeed, the hypothetical benefits do not outweigh the contamination risk for operators. The risk/benefit ratio should be evaluated on a single-case basis in patients with bronchiectasis or with evident bronchial encumbrance using tools at a safe distance from the patient, which can be maintained.

After discharge from intensive care or an intermediate care, patients may present with disability and functional damage (respiratory function, critical illness myopathy, and neuropathy), reduced participation, and deterioration of quality of life, either in the short- and long-term following discharge. Recovery time is variable depending on the degree of normocapnic respiratory failure and associated physical (asthenia, peripheral muscle weakness) and emotional (anxiety, depression, sense of abandonment, post-traumatic stress syndrome) dysfunction (55). Comorbidities make longer the return to the former condition. Evaluation of exercise capacity and oxygenation response on effort (by the 6-min walk test) and at nighttime should be planned as soon as possible. For patients bedridden for extended periods, an assessment of balance function is especially recommended. Further suggestions include: evaluation of peripheral muscle strength by the MRC scale, measurement of joint range-of-motion (ROM), and manual and isokinetic muscle tests. Simple and repeatable treatment protocols for weaning patients from oxygen therapy are indicated. Reconditioning interventions are advised in weaned patients and those requiring prolonged weaning from mechanical ventilation and oxygen use, to improve the physical status and to rebalance the motor, and cognitive consequences of prolonged immobilization (72, 98). Exercise involving a gradual load increase is recommended to regain normal function. Low intensity exercise (<3.0 metabolic equivalents), daily patient counseling, and education are urged. Patients discharged home or to other facilities in the community should receive instruction on physical activity plans. These must be closely monitored regarding function, capacity, and participation once the patient is no longer contagious.

Concerning tracheotomy weaning, our experience emphasizes the importance of patient positioning (head in high flexion) and regular tracheostomy care (cleaning the stoma, changing the inner cannula, aspirations). We use the Facial Oral Tract Therapy (FOTT®) concept and patient positioning according to the Bobath® concept (99) as stimulation techniques that we start immediately on patient admission. Deflation of the cuff is performed during treatment sessions, as soon and as often as possible, with the longest permissible duration, depending on the patient's tolerance. Cuff deflation, even in patients with altered consciousness, avoids deafferentation of the oropharyngeal region. The cuff is inflated during respiratory physiotherapy when ventilation is required and humidification is constantly provided. An appropriate stimulation (cuff deflation, stimulation of upper airway respiration, swallowing, coughing, and verbal communication) it's helpful to avoid sensory deafferentation. Physicians and physiotherapists must work closely with the Ear Nose and Throat (ENT) specialists. The timing of the first trans-cannulation depends on the type of tracheostomy in question. Use of the open surgical approach is recommended between the second and fifth days while the percutaneous dilatational approach is favored after 10 days. A fenestrated outer cannula is inserted at this moment, with the cuff deflated to allow air to flow over the vocal folds when the orifice is plugged by a finger, speaking valve, or stopper. The stimulation from airflow passing over the vocal cords is essential for laryngeal re-afferentation. When saliva-flow management seems to be safe and ventilation treatment is no longer need, the tracheostomy tube can be removed.

Several studies have confirmed that intervention by a multidisciplinary team reduces weaning time (47, 99, 100). Although several individualized, non-comparative, and non-validated decannulation protocols exist, there is no universally accepted protocol. Additionally, randomized clinical trails are lacking on this critical issue. However, our group has demonstrated the benefits of the interdisciplinary neurosensory weaning program in a retrospective study. It showed a reduction in weaning failure rate from 27 to 9%. Furthermore, the time to decannulation after admission decreased from 19 to 12 days (101).

Early and intensive treatments conducted by a specialized team reduce the complications associated with bed rest therefore improving patient outcomes (102). Defining specific guidelines for individual patient pathways will enable creating treatment plans suitable for multiple settings (75). Ineffective cough and secretion retention can play a significant role in weaning failure. In this respect, evaluation of cough strength by peak expiratory flow rate can predict extubation failure and may reduce the length of ICU stay and as a result, costs, morbidity, and mortality may also decrease. Cough stimulation techniques, including lung volume recruitment or manually and mechanically assisted cough are used to facilitate extubation and prevent post-extubation respiratory failure. However, the sub-standard quality of studies on this topic make it difficult to draw conclusions regarding the effectiveness of the techniques (103).

## Conclusions

Although our understanding of COVID-19 is still incomplete, a growing body of evidence indicates potential deleterious effects on CNS and PNS function. This may lead to complex and long-lasting physical, cognitive, and functional impairments. Beginning rehabilitation in the acute stage of the disease is required to combat this.

COVID-19 is associated with a cascade of negative concurrent factors, including some unrelated to the disease *per se*, all having a potentially heavy impact on disability and global functioning. This additive effect with ability to induce multi-organ dysfunction is peculiar to COVID-19 and differentiates it from CNS, PNS, heart and lung diseases for example, which, even when very severe, rarely display such a pleiotropic effect. Patients with severe COVID-19 are likely therefore, to present with a variety of serious sequelae associated with the viral illness, including prolonged stay in the ICU, immobilization, mechanical ventilation, tracheotomy, sedation, delirium, all aggravated by preexisting comorbidities.

Given the high proportion of hospitalization in critical care units, it is likely that a considerable number of survivors will require rehabilitation due to these sequelae. Hence, rehabilitation will be a key component in the continuum of patient-centered care and rehabilitation professionals will have a critical role in assisting patient recovery from COVID-19-associated disabling effects. Indeed rehabilitation by a multidisciplinary team should start as early as possible since prompt intervention has proven efficient in counteracting the vicious circle of disease-related and indirect ICU side-effects. Accordingly, individualized treatment plans should be implemented.

Based on the experience of our acute interdisciplinary neuro-rehabilitation team in managing severely brain-injured patients, we would recommend applying an early and intensive rehabilitation program for severe COVID-19 patients that aims at maximizing patient function to achieve the highest possible level of independence (Figure 2). Such programs consist of a combination of approaches including early mobilization, multimodal sensory, and cognitive stimulation, tracheotomy-weaning strategies, cardiovascular training and monitoring and respiratory management. These have been shown to improve functional outcomes and quality of life, reduce the social and emotional burdens for the patient and family, and reduce the length of hospitalization and related costs.

## Author Contributions

AP, JJ, LL, LD, PR, NS, and KD contributed to the conception and design of the review. AP, JJ, and LP contributed to the literature review, drafting the text, and preparing the figures. All authors contributed to the article and approved the submitted version.

## Conflict of Interest

The authors declare that the research was conducted in the absence of any commercial or financial relationships that could be construed as a potential conflict of interest.
